# High-speed multifocal plane fluorescence microscopy for three-dimensional visualisation of beating flagella

**DOI:** 10.1242/jcs.231795

**Published:** 2019-08-15

**Authors:** Benjamin J. Walker, Richard J. Wheeler

**Affiliations:** 1 Wolfson Centre for Mathematical Biology, Mathematical Institute, University of Oxford, Oxford OX2 6GG, UK; 2 Peter Medawar Building for Pathogen Research, Nuffield Department of Medicine, University of Oxford, Oxford OX1 3SY, UK

**Keywords:** Cilia, Flagella, *Leishmania*, Microscopy, *Trypanosoma*

## Abstract

Analysis of flagellum and cilium beating in three dimensions (3D) is important for understanding cell motility, and using fluorescence microscopy to do so would be extremely powerful. Here, high-speed multifocal plane fluorescence microscopy, where the light path is split to visualise multiple focal planes simultaneously, was used to reconstruct *Trypanosoma brucei* and *Leishmania mexicana* movement in 3D. These species are uniflagellate unicellular parasites for which motility is vital. It was possible to use either a fluorescent stain or a genetically-encoded fluorescent protein to visualise flagellum and cell movement at 200 Hz frame rates. This addressed two open questions regarding *Trypanosoma* and *Leishmania* flagellum beating, which contributes to their swimming behaviours: 1) how planar is the *L. mexicana* flagellum beat, and 2) what is the nature of flagellum beating during *T. brucei* ‘tumbling’? We showed that *L. mexicana* has notable deviations from a planar flagellum beat, and that during tumbling the *T. brucei* flagellum bends the cell and beats only in the distal portion to achieve cell reorientation. This demonstrates high-speed multifocal plane fluorescence microscopy as a powerful tool for the analysis of beating flagella.

## Abstract (precis)

**Summary:** A new 3D fluorescence microscopy-based method allows analysis of the high speed beating of cilia and flagella, with potential to answer open questions about *Leishmania* and *Trypanosoma* 3D motility.

## INTRODUCTION

**Fig. 1.**
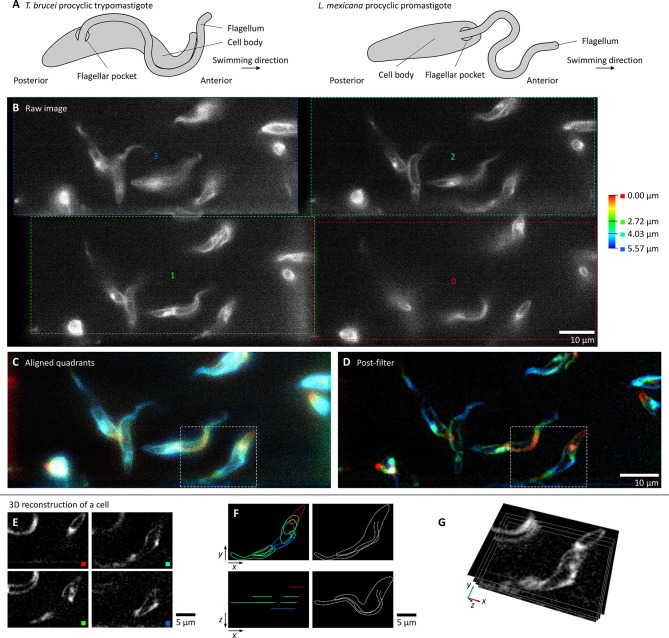
**High-speed multifocal plane microscopy to visualise live swimming *T. brucei* in 3D.** All micrographs show a single frame from a 200 Hz video  of procyclic forms labelled with FM 4-64FX (Movie 1). (A) Schematic representation of the morphology of *T. brucei* and *L. mexicana* procyclic forms. (B) The raw camera image, showing the four sub-images (0–3) and their focal offsets. (C) The image in B, following sub-image alignment and pseudocolouring according to focal depth. (D) The image in C following filtering. (E–G) 3D reconstruction of an example cell, outlined in white box in C and D, showing individual focal planes (E), membrane contours from each focal plane (left) and the 3D conformation (right) (F), and direct visualisation in 3D (G).

Trypoanosomatid parasites, including the human pathogens *Trypanosoma brucei*, *Trypanosoma cruzi* and *Leishmania* spp., have a single flagellum whose motility is vital for progression through the life cycle (Beneke et al., 2019; Broadhead et al., 2006; Rotureau et al., 2013; Shimogawa et al., 2018). Different species and life cycle stages have linked morphological and motility adaptations, and trypanosomatid morphologies are defined, in part, by the flagellum that is laterally attached to the cell body along most of its length in the trypomastigote morphology, for a shorter portion of its length in the epimastigote and simply protrudes from the cell anterior in the promastigote (Hoare and Wallace, 1966) (Fig. 1A).

*Trypanosoma brucei* trypomastigotes, including procyclic (fly midgut) and mammalian bloodstream forms, have a complex three-dimensional (3D) cell movement (Bargul et al., 2016; Heddergott et al., 2012; Rodríguez et al., 2009; Schuster et al., 2017; Wheeler, 2017). They can swim in liquid, but the fly gut and salivary glands can be densely packed with parasite cells (Schuster et al., 2017). When they are free in a volume of liquid, such as the bloodstream, they swim far slower than the fluid flow (Heddergott et al., 2012; Krüger et al., 2018), yet they can invade tissues (Capewell et al., 2016; Trindade et al., 2016). Taken together, this suggests complex motility functions, which includes collective motion of densely packed cells (Eliaz et al., 2017; Imhof and Roditi, 2015; Oberholzer et al., 2010; Saada et al., 2015) and surface-bound antibody clearance (Cheung et al., 2016; Engstler et al., 2007). *Leishmania* promastigote and *T. cruzi* epimastigote swimming is somewhat unlike *T. brucei* (Ballesteros-Rodea et al., 2012; Gadelha et al., 2007; Wheeler, 2017), and the function of their motility is less well-analysed but is probably also complex (Forestier et al., 2011). As for *T. brucei*, they will also probably undergo hydrodynamic coupling when densely packed, which could give collective population movement at the macro scale.

Contributing to complex motility are at least two modes of flagellum movement. *T. brucei*, *T. cruzi*, *Leishmania* and related organisms normally undergo a tip-to-base symmetrical flagellar-type beat (Holwill, 1965; Walker, 1961). In *Leishmania* promastigotes and *T. cruzi* epimastigotes this is near-planar (Gadelha et al., 2007; Holwill and McGregor, 1975; Wheeler, 2017) and at ∼20‒25 Hz in *Leishmania* (Gadelha et al., 2007; Wheeler, 2017). They can also switch to a base-to-tip beat (Ballesteros-Rodea et al., 2012; Edwards et al., 2018; Gadelha et al., 2007; Heddergott et al., 2012; Holwill and McGregor, 1975). In *T. cruzi* epimastigotes and *Leishmania* promastigotes this reversed beat is an asymmetrical ciliary-type beat at lower frequency [6±1 Hz (mean±s.e.m.) in *L. major*] and causes cell reorientation (Ballesteros-Rodea et al., 2012; Edwards et al., 2018; Gadelha et al., 2007). The function of beat type-switching is not yet clear; however, persistent base-to-tip beating in *Leishmania* (upon deletion of the distal outer dynein arm docking complex protein dDC2; LmxM.31.2900) (Edwards et al., 2018) prevents transmission through the sandfly vector (Beneke et al., 2019). In *T. brucei* the tip-to-base beat occurs at ∼15‒20 Hz (Branche et al., 2006; Heddergott et al., 2012; Rodríguez et al., 2009; Schuster et al., 2017; Wheeler, 2017). The reversed beat is not so well characterised [except in mutants (Baron et al., 2007; Branche et al., 2006)], but occurs at a lower frequency [∼3‒5 Hz in procyclic forms (Branche et al., 2006), 13.1±0.8 in bloodstream forms (Heddergott et al., 2012)] with a higher amplitude. It causes slow backwards swimming or ‘tumbling’ (Heddergott et al., 2012), a term used to describe rapid cell reorientation. Again, switching to a reversed beat has no clear function, but different *T. brucei* life cycle stages in the tsetse fly vector have different propensities for tumbling (Schuster et al., 2017).

Previous analyses of trypanosomatid cell and/or flagellum movement have been two-dimensional, with three exceptions for *T. brucei*. One is qualitative, defocus of bright-field microscopy to infer distance from the focal plane (Heddergott et al., 2012). Two are quantitative, an optical tomography approach (Heddergott et al., 2012) and mathematical model fitting (Wheeler, 2017). However, both quantitative methods required a perfectly repeating beat and constant cell rotation rate. More broadly, three main approaches have been used to perform 3D flagellar beat analysis. First, inferring defocus distance using bright-field microscopy (Bukatin et al., 2015), which is limited to semi-quantitative depth information. Second, using a high-frequency oscillating objective (rapid focus change) to visualise multiple focal planes (Silva-Villalobos et al., 2014). Here, focal planes capture is only near-synchronous, leading to a 3D analogue of rolling shutter artefacts. Objective motion will also give blurring in *z*. Third, high-speed digital holographic microscopy, as used for sperm (Daloglu et al., 2018) and *Plasmodium* microgametes (Wilson et al., 2013). This has relatively low signal-to-noise and requires complex image analysis. These approaches have only been used ‘label free’ using transmitted light illumination – indeed, inferred defocus distance and digital holographic microscopy can only be used with transmitted light.

Multifocal plane fluorescence microscopy (Gan et al., 2013; Prabhat et al., 2007) has the potential to achieve the frame rates and sensitivities necessary for analysis of flagellum beating in 3D, with the advantage of high fluorescent label specificity. Here, it was applied to open questions concerning *T. brucei* and *L.* *mexicana* motility. First, using membrane stain FM 4-64FX to analyse *T. brucei* flagellum beating during tumbling, which has not previously been analysed in 3D. This showed that tumbling arises from reversed distal flagellum beating while the proximal flagellum ‘locks’ the cell in a curved configuration that may arise from proximal and/or distal differences in axoneme (particularly outer dynein arm) composition (Edwards et al., 2018; Subota et al., 2014). Second, a fluorescent protein fused to a flagellar membrane protein at the endogenous locus was used to quantify *Leishmania* beat planarity, previously assumed to be near-planar. This showed that flagellum bending remains within a plane, but that the plane can change over a beat cycle. This requires twisting at the flagellum base, probably linked with flagellum attachment in the pocket (Wheeler et al., 2016) or factors specific to the proximal axoneme (Edwards et al., 2018). *Leishmania* have a tendency to swim in curved paths (Wheeler, 2017) and this phenomenon may be responsible.

## RESULTS AND DISCUSSION

Multifocal plane imaging was initially tested with *T. brucei* labelled with FM 4-64FX in normal growth medium. *T. brucei* movement deviates significantly from a single plane and requires 100 Hz or faster frame rates to effectively ‘freeze’ flagellum/cell movement for analysis. Multifocal plane imaging was configured using a light path multi-splitter with three semi-silvered mirrors, which split the light to four quadrants of a single camera, and lenses at the pupil planes to introduce focus offsets (Fig. S1A). Lens focal lengths were selected for a ∼2 µm focal plane (*z*) spacing and the actual focus offset and the sub-image position on the camera sensor (in *x* and *y*) were calibrated using fluorescent beads (Fig. S1). Sub-image image position and magnification aberration was measured and corrected (Fig. S1B,C,D), allowing precise sub-images alignment into a final *z* stack.

200 Hz frame rates were achieved with a 2560×1080 px image resolution. At four focal planes this corresponds to ∼0.5 gigavoxel/second with ∼8 photons/px background and signal up to ∼50 photons/px (Fig. 1B; Movie 1A). Sub-image alignment and colour coding by focal plane offset gives a 3D view of cell conformation (Fig. 1C; Movie 1B) and image filtering highlights the in-focus membrane at each focal plane (Fig. 1D; Movie 1C), effectively capturing *T. brucei* morphology (Fig. 1E). The cell and flagellum membrane contours can be interpreted to give a plausible 3D configuration, including the position of the flagellum and flagellar pocket at its base (Fig. 1F). Alternatively, the data can be directly visualised in 3D (Fig. 1G).

### Flagellum beat during *T. brucei* tumbling

*T. brucei* ‘tumbling’ associated with beat reversal occurs commonly in the metacyclic forms in tsetse fly salivary glands (Schuster et al., 2017) and occasionally in other life cycle stages (Branche et al., 2006; Heddergott et al., 2012; Uppaluri et al., 2011). While it has no unambiguous function, understanding tumbling is valuable as it causes cell reorientation that could allow a ‘run and tumble’-like chemotaxis mechanism. *T. brucei* experience a low Reynolds number environment where reversal of the flagellum beat for forward swimming would simply reverse swimming direction (Purcell, 1977). Therefore, tumbling must also involve some other change; perhaps an unrecognised asymmetry in the reversed *T. brucei* beat or perhaps, as previously noted (Heddergott et al., 2012), a difference in motion between the proximal and distal flagellum. Analysing tumbling cells is challenging as they have a ‘less straight’ configuration (Uppaluri et al., 2011) that sits poorly in a focal plane.

**Fig. 2.**
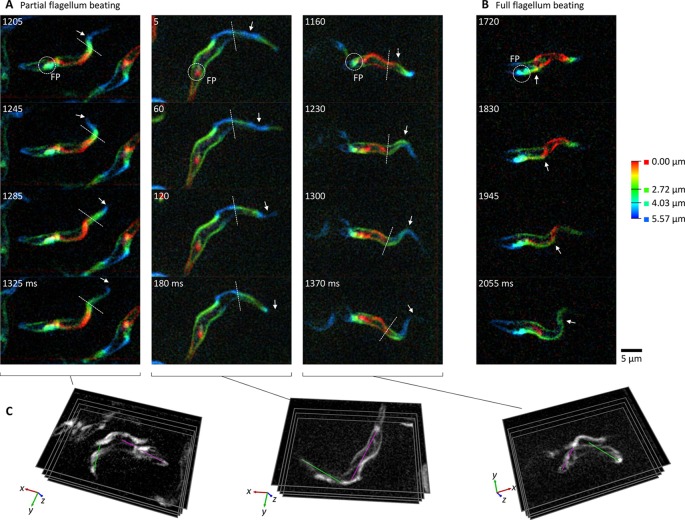
**The distal flagellum beat reverses while the proximal flagellum curves the cell in tumbling *T. brucei*.** All panels show frames from 200 Hz multifocal plane videos of procyclic forms labelled with FM 4-64FX (Movie 2). (A) Three examples of typical tumbling cell movement showing four frames covering one beat at ∼5 Hz. FP indicates the flagellar pocket, arrows indicate a propagating wavefront, dotted lines indicate the boundary between the beating and ‘locked’ flagellum portions. (B) One example of the less common flagellum movement in tumbling cells where waves propagate along the entire flagellum. (C) 3D conformation of the cells in A highlighting the orientation of the cell anterior (green line) or posterior (magenta line) of the locked portion of the flagellum, which lie approximately perpendicular.

To characterise tumbling, procyclic form cells were labelled with FM 4-64FX and placed in a sample chamber. Tumbling cells sedimented near the coverslip and 200 Hz multifocal plane videos showed that they all underwent a reversed beat; however, in the majority (75%, *n*=28) the proximal half of the flagellum did not move. Instead, the waveforms initiated in the middle of the flagellum and propagated towards the tip (Fig. 2A; Movie 2A). In the remainder, the entire flagellum moved and the waveforms initiated near the flagellum base (Fig. 2B; Movie 2B). Tumbling was similar to the motion of outer dynein arm mutants following RNAi knockdown of intermediate (DNAI1) (Branche et al., 2006) or light (LC1) (Baron et al., 2007) chains. This further supports the hypothesis that beat reversal in *T. brucei* involves changes to outer dynein arm regulation.

Forward-swimming cells under the same conditions underwent a tip-to-base flagellum beat while rotating around their long axis (Fig. S2). This matches previous analysis by optical tomography (bloodstream forms) (Heddergott et al., 2012) and mathematical model fitting (procyclic forms) (Wheeler, 2017), and is incompatible with a bihelical beat (Rodríguez et al., 2009).

In tumbling cells where flagellum movement was restricted to the distal half of the flagellum (the cell anterior), the proximal half of the flagellum (the cell middle) was ‘locked’ in a curve on a ∼100 ms timescale (Fig. 2A; Movie 2A). Viewing these cells in 3D (Fig. 2C) showed a ∼90° bend in the middle of the cell that was not always clear from 2D images. The distal flagellum beat plane was perpendicular to the plane in which the proximal flagellum bent the cell, consistent with a helical attachment of the flagellum to the cell body. The impact of cell curvature on swimming was simulated using a boundary element computational framework, which showed a 90° mid-cell bend that causes a circular swimming path with a radius less than the cell length (Fig. S3), qualitatively similar to reorientation while tumbling. The flagellum in tumbling cells is therefore responsible for a beat and cell configuration that are not a time reversal of the normal tip-to-base beat, leading to rapid reorientation and explaining why tumbling cells appear less straight.

For forward swimming, waveforms initialise at the flagellum tip, while, for tumbling, flagellar waveforms tend to initialise mid-flagellum rather than at the flagellum base. Sites conducive for waveform initiation may be defined by proximal and/or distal differences in the molecular composition of outer dynein arms (Edwards et al., 2018) or other axoneme components (Subota et al., 2014). Alternatively, as cell body and flagellum movement are tightly coupled by the flagellum attachment zone and the attached cell body is wider towards the flagellum base, it may be too rigid (Sun et al., 2018) for the reversed beat to bend.

### Procyclic *L. mexicana* beat planarity

**Fig. 3.**
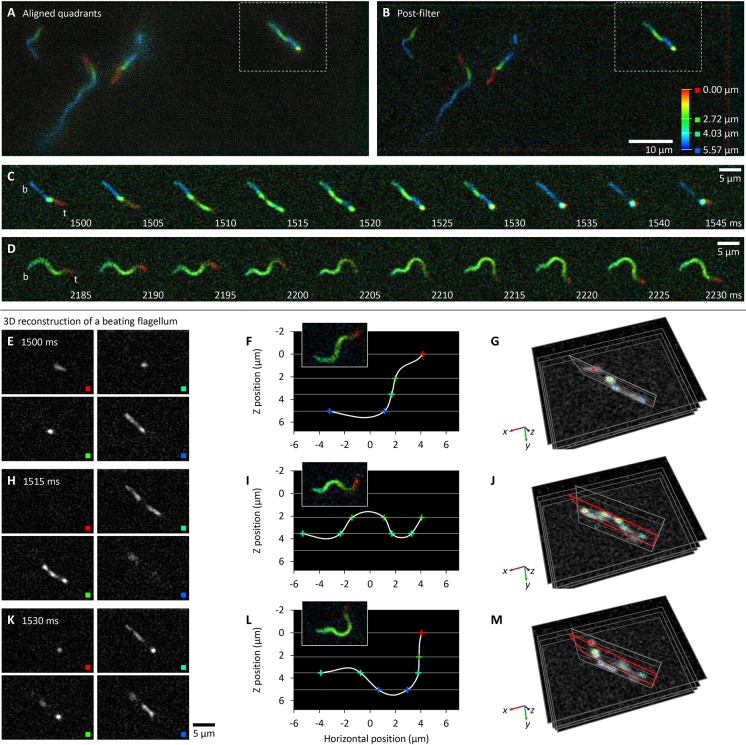
**The *L. mexicana* flagellar beat does not remain in a single plane.** All panels show frames from a 200 Hz multifocal plane video of promastigotes expressing SMP1::mNG (Movie 3). (A) One example frame, following sub-image alignment and pseudocolouring according to focal depth. (B) The image in A following filtering. (C,D) Movement of an example flagellum, outlined by white box in A and B, beating at ∼20 Hz. Labels indicate the flagellum base (b) and tip (t). (C) One beat when the beat and focal planes are perpendicular. (D) One beat from the same cell, 0.7 s later, when the beat and focal planes are parallel. (E–M) 3D flagellum conformation from three frames (1500 ms, 1515 ms and 1530 ms in C). E,H,K show individual focal planes. F,I,L show plotted points of intersection of the flagellum with each focal plane. Inset, a rotated copy of 2185 ms, 2200 ms and 2215 ms, respectively, in D for comparison. (G,J,M). Direct visualisation in 3D, with J and M showing the plane in G in red.

*Leishmania* (and related species) have a near-planar flagellum beat but have only been analysed when confined to a thin layer (Gadelha et al., 2007; Holwill and McGregor, 1975; Wheeler, 2017). Evidence for planarity – that the whole flagellum appears in focus when the beat and focal planes are parallel – is also qualitative and limited by microscope *z* resolution. To address this, *L. mexicana* promastigotes expressing mNeonGreen (mNG) fused to the flagellum membrane protein SMP1 were analysed by multifocal plane microscopy, focusing away from the coverslip to avoid surface effects. A 200 Hz frame rate could be achieved, with <1 photon/px background and signal up to 15 photons/px (Fig. 3A,B; Movie 3A,B).

Most *Leishmania* underwent a tip-to-base symmetrical flagellar beat with the beat plane oriented randomly relative to the focal planes. A few cells rotated sufficiently quickly that, within a single video (4.75 s), their beat plane rotated from near-parallel to near-perpendicular, giving orthogonal views of a single cell (Fig. 3C,D). The 3D flagellum conformation was reconstructed by recording where it intersected with a focal plane while beating near-perpendicular to the focal planes (Fig. 3E–M). By considering a cell that rotates over the video, the beat reconstruction from multifocal plane imaging can be compared to widefield imaging once it has rotated parallel to the focal plane (Fig. 3F,I,L). This confirmed accurate 3D reconstructions.

The points of flagellum–focal plane intersection can be precisely measured as the computational sub-image alignment has a small error (Fig. S1D). Flagellum beat planarity was quantified from frames where the flagellum crossed the focal planes at ≥5 points. These were fitted to a plane and planarity inferred from the residuals – the distance of points from the fitted plane. Overall, residuals were small (*n=*23 reconstructions from nine cells), indicating that the flagellum in any single frame was very close to planar. The mean residual (overall deviation from the plane) was 0.076±0.058 µm, while the maximum residual (maximum deviation from planarity at any single point) was also small at 0.32±0.24 µm. However, while the flagellum configuration was near-planar in individual frames, this plane oscillated over a beat cycle, particularly for short flagella (illustrated in Fig. 3C,D).

Subtle flagellum beat deviations from planar in sperm is important for cell swimming (Bukatin et al., 2015), therefore *Leishmania* deviation from planarity is probably important, perhaps for their characteristic helical swimming paths (Wheeler, 2017). *Leishmania* experience a force- and torque-free low Reynolds number environment, therefore there must be an asymmetric cell body motion to allow flagellum beat plane rotation. This implicates the flagellum–cell body interface as giving rise to variation in the beat plane, perhaps proximal flagellum-specific outer dynein arm complexes (Edwards et al., 2018) or alternatively, flagellum anchoring in the flagellar pocket by the flagellum attachment zone (Wheeler et al., 2016).

### Conclusions

This work demonstrates that multifocal plane fluorescence microscopy is capable of analysing volumes and frame rates useful for interrogating flagellum beating using either chemical or native fluorescent protein fluorescence. This is, to our knowledge, the highest frame rate biological application of this approach and the first 3D reconstruction of flagellum beating using fluorescence. Analysis of *T. brucei* tumbling and *L. mexicana* forward swimming approach highlighted how small changes in cell movement or configuration (a curved cell shape in *T. brucei* or a varying beat plane in *L. mexicana*) alter cell swimming, which may be important for collective motility or chemotaxis. It also further emphasised how the coupling of flagellum movement to the cell body by the flagellum attachment zone and how proximal and/or distal differences in the outer dynein arms may regulate trypanosomatid swimming.

The approach demonstrated here is transferrable to other species or ciliated and flagellated tissue, and specificity of fluorescence labelling provides key advantages over transmitted light used in digital holography and bright-field defocus methods. There are also more complex applications, for example a labelled cell crowded by unlabelled tissue or other cells – in trypanosomes such conditions are where collective motility occurs. Labelling of a specific cellular substructure is also powerful, particularly for complex multi-flagellated cells like *Giardia* and *Trichomonas*. High-speed multifocal plane microscopy is therefore a powerful approach for understanding flagellum- and/or cilium-driven dynamics.

## MATERIALS AND METHODS

SmOxP9 procyclic *T. brucei* (Poon et al., 2012) were grown in SDM79 medium (Life Technologies) with 10% FCS. Cas9T7 promastigote *L. mexicana* (Beneke et al., 2017) were grown in M199 medium (Life Technologies) supplemented with 2.2 g/l NaHCO_3_, 0.005% hemin, 40 mM HEPES-HCl (pH 7.4), and 10% FCS. *L. mexicana* SMP1 (LmxM.20.1310) (Tull et al., 2004) was C-terminally tagged with mNG (Shaner et al., 2013) at the endogenous locus as previously described (Beneke et al., 2017). *T. brucei* and *L. mexicana* were grown at 28°C and imaged during logarithmic growth (0.5×10^7^‒1.0×10^7^ cells/ml).

Videos were captured using a 100× NA 1.4 objective (without phase ring, Zeiss) on an Axio observer A1 (Zeiss) microscope with incubator chamber, using a 120 V metal halide light source (Zeiss, HXP 120 V) and either an mRFP (Zeiss, 63HE) or GFP (ThorLabs, MDF-GFP2) filter cube. A MultiSplit v2 (Cairn Research) was used for multifocal plane imaging with three 50% semi-silvered mirrors in the filter cubes and a 2000 mm, 1300 mm, 500 mm or no lens in the auxiliary filter/lens mount immediately following the filter cube.

Images for calibration of focus offset, position offset and scale/magnification aberration of each sub-image were captured using multi-wavelength fluorescent beads (TetraSpeck, Invitrogen T7279). Focus offset was measured from the relative *z* position to focus the beads in each sub-image. Sub-image position offset and scale aberration were determined from bead pairs (one central and one peripheral bead within each sub-image) using Gaussian fitting to determine bead locations with sub-pixel accuracy.

Cells were held in a sealed 250 µm deep (deep relative to cell size) sample chamber (Gene Frame, Thermo Fisher Scientific, AB0576) between a plain glass slide and coverslip in normal growth medium and analysed at 28°C. For FM 4-64FX (Thermo Fisher Scientific Molecular Probes, F34653) labelling at 1:100, 40 µM of the stain was included in the medium.

Videos at a length of 4750 ms at 200 Hz frame rate (5 ms/frame) were captured using a Neo 5.5 camera (Andor), vertically cropped to 50% (1080 px) around the camera midline. Constant readout column and pixel noise were subtracted (mean signal from 600 frames captured with no illumination) and random amplifier noise per row of pixels was subtracted (the median signal per pixel row per frame). Focus stacks were then generated using the necessary offset and scale corrections. Image filtering for analysis was a 2 px Gaussian blur then a 10 px rolling ball background subtraction. Image processing was performed in ImageJ (Collins, 2007).

Cell swimming was simulated using a boundary element computational framework (Pozrikidis, 2002) of a neutrally buoyant mesh approximating a procyclic *T. brucei* cell shape, essentially as previously described for *Leishmania* simulation (Walker et al., 2018; Walker et al., 2019). The *T. brucei* cell mesh was approximated from three joined tapering cylinder sections and capped with hemispheres. From anterior to posterior, written in the form (distance from anterior, diameter), cell shape was parameterised as: (0 µm, 1 µm), (6 µm, 3 µm), (12 µm, 2 µm), (18 µm, 0.5 µm). The flagellum beat was simulated as a base-to-tip sinusoid with amplitude 1.5 µm in the cell anterior (12‒18 µm) and cell curvature was approximated as a circular arc in the cell mid-section (6‒12 µm) to achieve a 0°, 45° or 90° cell bend.

## Supplementary Material

Supplementary information
