# Supplementary material for: High-speed multifocal plane fluorescence microscopy for three-dimensional visualisation of beating flagella
Source: J Cell Sci. 2019 Aug 15;132(16):jcs231795. doi: 10.1242/jcs.231795 (PMC6737910; doi:10.1242/jcs.231795)
Supplement: Supplementary information [file joces-132-231795-s1.pdf]

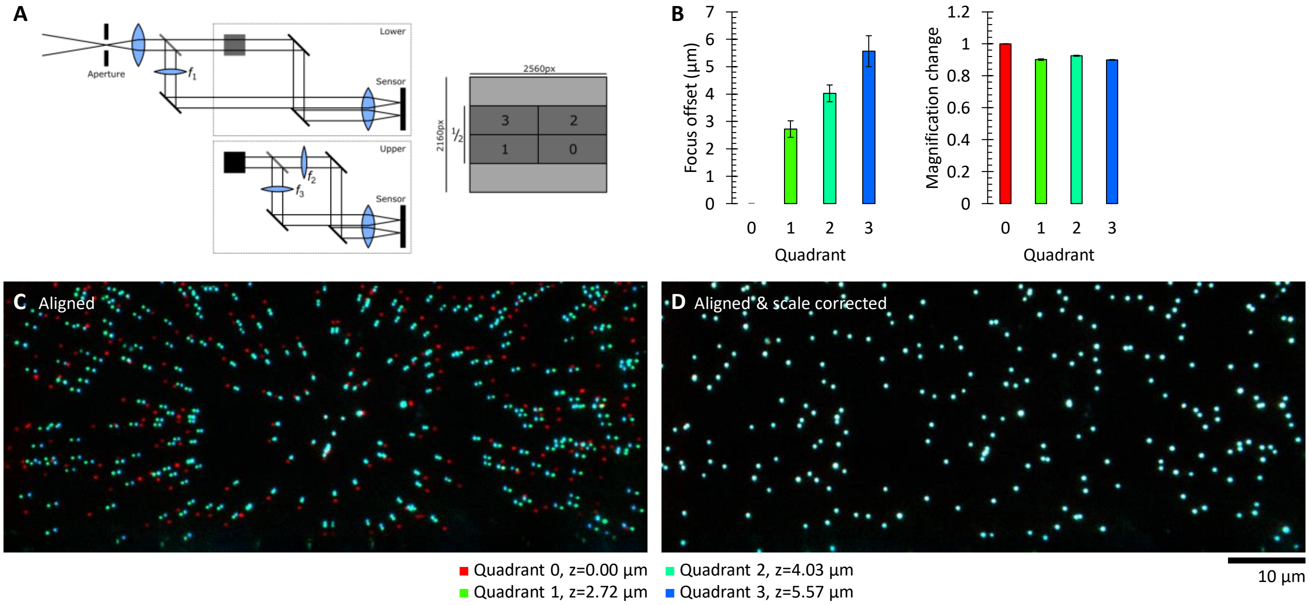

Fig. S1. Setup and calibration of the multifocal plane system. A. Overview of the light path through the multi-splitter to achieve images of the same region but with offset focal planes on four quadrants of the camera sensor. Semi-silvered mirrors are shown in grey, fully-silvered in black. The light path is 3D, with the grey square (representing a semi-silvered mirror) in the lower section reflecting light upwards, out of the plane of the page/screen, and the black square (representing a fully-silvered mirror) in the upper section reflecting it back to parallel with the page/screen. Only half (full width, vertically centred) of the camera sensor was used, allowing a 200 Hz frame rate. f_1 = 2000 mm, f_2 = 1300 mm, f_3 = 500 mm. **B.** The measured offset in focal plane for the four quadrants and the measured scale/magnification aberration for the four quadrants relative to the light path with no additional lens. Error bars represent the standard deviation, n = 5. **C.** Example of images aligned from the four quadrants (captured at the appropriate stage height for the beads to be in focus) without correction for the scale/magnification aberration. **D.** The same set of images aligned from the four quadrants with scale/magnification correction, showing precise co-localisation. Offset and scale parameters were accepted if the mean offset between points from different channels was <1 px (<65 nm).

Journal of Cell Science • Supplementary information

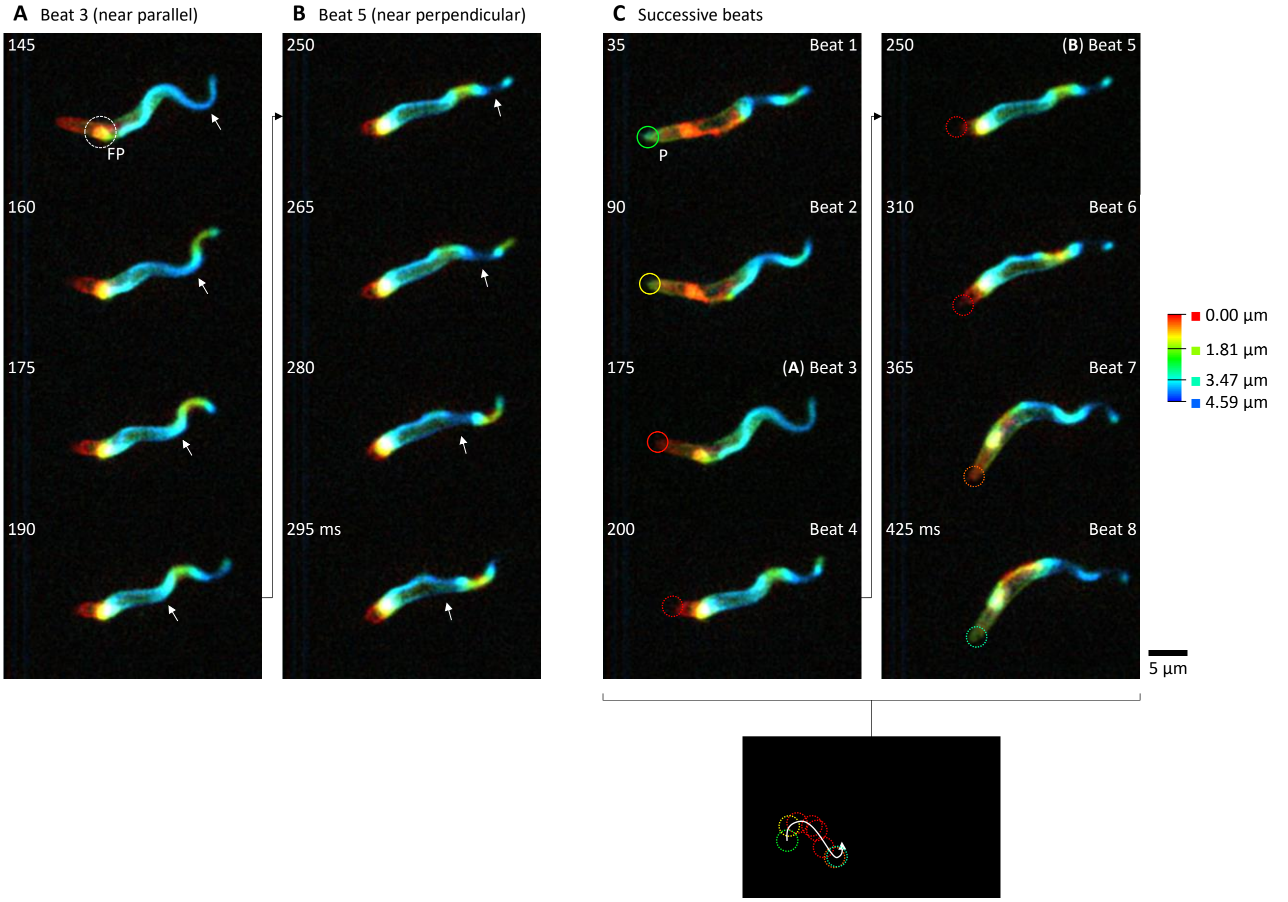

Fig. S2. Forward swimming *Trypanosoma brucei* undergo a tip-to-base whole-flagellum beat while undergoing longitudinal rotation, as previously described. All panels show frames from a 200 Hz multifocal plane videos of a *T. brucei* procyclic form labelled with a fluorescent membrane stain. **A.** Example of the flagellum movement in a forward swimming *T. brucei* procyclic cell showing four frames covering one beat cycle (beat frequency ~20 Hz) while the flagellum beat plane is near parallel to the focal planes. Waves propagate from the flagellum tip to the base along the entire flagellum. FP indicates the flagellar pocket at the base of the flagellum, arrows indicate a propagating wavefront. **B.** Four more frames of the same *T. brucei* procyclic form cell as in A showing one beat cycle where the cell has rotated such that the flagellum beat plane is near perpendicular to the focal planes. **C.** The appearance of the same *T. brucei* procyclic cell at the start of the beat cycle for 8 successive flagellar beats. P indicates the posterior of the cell, whose position as the cell swims forward is summarised at the bottom of panel C. The cell completes one longitudinal revolution over the 8 beat cycles.

Journal of Cell Science • Supplementary information

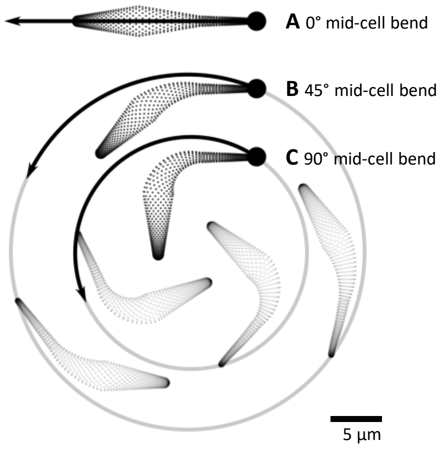

Fig. S3. Simulated paths of procyclic *T. brucei*-shaped cells driven by beating approximating observed tumbling cells. Swimming path of a mesh approximating the shape of the procyclic *T. brucei* cell moving in a low Reynolds number environment driven by a sinusoid beat in the anterior third of the cell, which approximates beating in only the distal half of the flagellum, and with different degrees of bending in the mid-cell, which approximates a locked flagellum curvature in the proximal half of the flagellum. The large circles indicate the start point of the anterior tip of the cell, the solid arrow indicates the path followed in a fixed time interval, and (for curved paths) the light grey line and ghosted cell shapes show the longer time scale behaviour. **A.** 0° bend in the mid-cell (straight cell). **B.** 45° bend in the mid-cell. **C**. 90° bend in the mid-cell, similar to the perpendicular orientation of the cell posterior and beating portion of the cell anterior observed for tumbling cells (Fig. 2).

Journal of Cell Science • Supplementary information

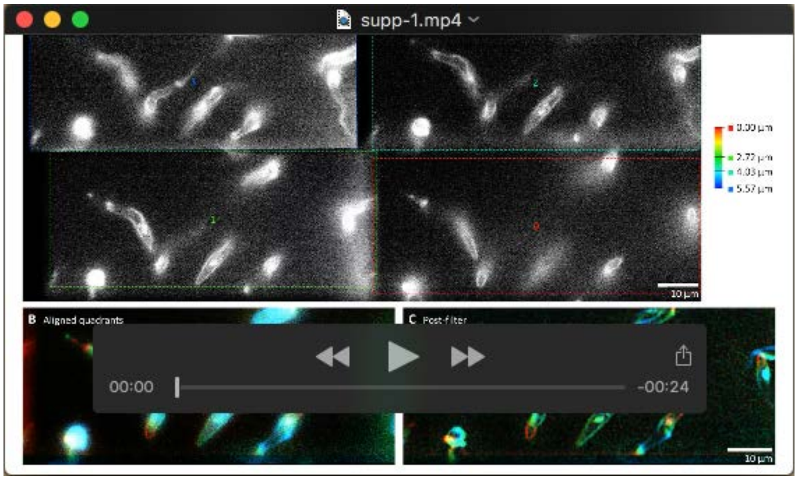

Movie 1. High-speed multifocal plane microscopy to visualise live swimming *T. brucei* in 3D. Animated version of Fig. 1, showing panels B-D. Playback is at 25 Hz frame rate (1/8 real time).

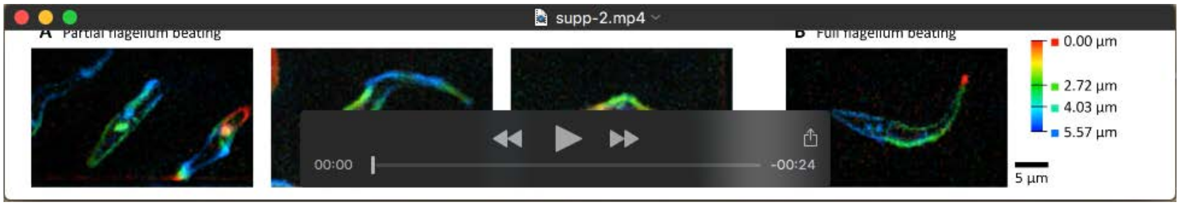

Movie 2. The distal flagellum beat reverses while the proximal flagellum curves the cell in tumbling *T. brucei*. Animated version of Fig. 2 showing panels A-B. Playback is at 25 Hz frame rate (1/8 real time).

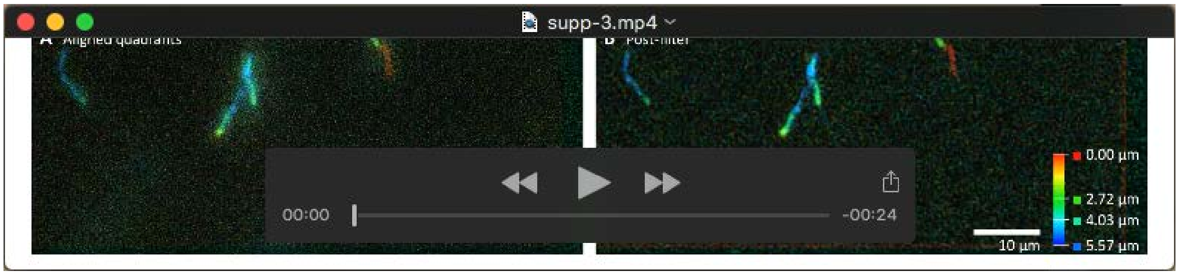

Movie 3. The *L. mexicana* flagellar beat does not remain in a single plane. Animated version of Fig. 3 showing panels A-B. Playback is at 25 Hz frame rate (1/8 real time).
